# Trends and Outcomes of US Hypertriglyceridemia-Induced Acute Pancreatitis Hospitalizations in Hispanic Americans: Epidemiology from 2016 to 2020

**DOI:** 10.1007/s40615-024-02171-1

**Published:** 2024-09-09

**Authors:** William S. Reiche, Ryan W. Walters

**Affiliations:** 1 Division of Gastroenterology and Hepatology, CHI Health Creighton University Medical Center, Omaha, NE 68124 USA; 2 Department of Clinical Research and Public Health, Creighton University School of Medicine, Omaha, NE 68124 USA

**Keywords:** Hypertriglyceridemia-induced acute pancreatitis, Disparities, Ethnicity, Cost, Plasmapheresis

## Abstract

**Objective:**

Hypertriglyceridemia-induced acute pancreatitis (HTG-AP) hospitalizations are increasing in the USA; however, the impact of race and ethnicity on key outcomes in Hispanic and non-Hispanic white HTG-AP hospitalizations has not been studied.

**Methods:**

We queried the National Inpatient Sample (NIS) between 2016 and 2020 identifying all patients with discharge diagnosis AP. HTG-AP hospitalizations were identified for Hispanic and non-Hispanic white patients. Primary outcomes included yearly rate of HTG-AP and in-hospital mortality from HTG-AP. Secondary outcomes were length of stay (LOS) and inflation-adjusted hospital costs.

**Results:**

HTG-AP hospitalizations accounted for 5.9% of all AP hospitalizations; 17,440 and 48,235 hospitalizations included a Hispanic and non-Hispanic white patient, respectively. The yearly rate of HTG-AP hospitalizations per 100,000 adult population was statistically higher for Hispanics compared to non-Hispanic whites. The HTG-AP hospitalization rate increased for both Hispanics and non-Hispanic whites (both *p*_trend_ < 0.001); however, the trends were not statistically different. The number of observed in-hospital deaths for Hispanics was too low to report, precluding subsequent analysis. Hispanics were younger, more likely to be female, more commonly Medicaid recipients, and from zip codes with lower income quartiles. Despite clinically similar rates of plasmapheresis use and LOS, adjusted hospital costs were 18.9% higher for Hispanics compared to non-Hispanic whites (95% CI, 15.4 to 22.6% higher, *p* < 0.001).

**Conclusions:**

HTG-AP incidence is increasing in the USA in Hispanic and non-Hispanic whites. Despite clinically similar outcomes, HTG-AP hospitalizations in Hispanic patients were associated with $26,805,280 in excess costs compared to non-Hispanic white hospitalizations.

## Introduction

Severe hypertriglyceridemia (HTG) is the etiology of acute pancreatitis (AP) in 2–5% of cases [1]. Severe HTG is defined as a serum triglyceride level greater than 1000 mg/dL and can be attributed to the cause of AP in the absence of other etiologies of AP. Severe HTG may be due to primary (gene mutations), secondary (alcohol or pregnancy), or a combination of primary and secondary etiologies [2]. Recent studies have found increases in AP and HTG-AP incidence [3–5]. This is concerning given HTG-AP often leads to a more severe clinical course, including more frequent organ failure compared to other etiologies [6].

Outcome differences associated with HTG-AP, race, and ethnicity have been infrequently studied. We found there are no prior studies of US populations comparing differences in cost and LOS data for HTG-AP hospitalizations in Hispanic and non-Hispanic whites. HTG is familial and confers a substantially increased risk for AP in patients with or without pregnancy [7]. Additionally, insulin resistance and HTG have been reported to vary with ethnicity and race [8, 9]. Moderate/severe HTG has been shown to be more prevalent in Hispanic patients than non-Hispanic whites. Given that these differences may represent health disparities, understanding social determinants will allow for physicians to provide more individualized care [10]. Provider awareness regarding the risk for initial or recurrent HTG-AP in patients with HTG can lead to focused initiatives to optimize therapy and target preventative efforts. If patients are aware of their risk and propensity for increased morbidity, patients may be incentivized to adopt preventative strategies.

We performed a retrospective review of the National Inpatient Sample (NIS) to investigate the incidence of HTG-AP hospitalizations from 2015 to 2020 and to determine the socioeconomic trends of patients hospitalized with HTG-AP. We aimed to study Hispanic and non-Hispanic white AP hospital hospitalizations to inform providers of trends in number of hospitalizations, length of stay (LOS), hospital cost, and characteristics of patients admitted for AP. We hypothesize that HTG-AP hospitalizations are becoming more frequent in patients with Hispanic ethnicity than non-Hispanic white patients. We also postulate Hispanic compared to non-Hispanic patients have different comorbidities, longer hospital stays, higher rates of readmission, and higher hospitalization costs due to multifactorial reasons.

## Materials and Methods

### Data Source

Hospitalization data were abstracted from the 2016–2020 NIS. The NIS was developed by the Health Care Cost and Utilization Project (HCUP) and is one of the largest inpatient databases in the USA that when weighted contains more than 35 million yearly discharges from HCUP-participating hospitals in 48 States and the District of Columbia [11–13].

### Hospitalization Cohort

We identified hospitalizations with a primary discharge diagnosis of HTG-AP in which the patient was Hispanic or non-Hispanic white and at least 18 years of age. Because there is no diagnosis code specific to HTG-AP, we identified HTG-AP using primary diagnosis codes specific to other or unspecified AP etiology (ICD-10-CM: K85.8 × and K85.9x, respectively) with a secondary diagnosis of HTG (pure hyperglyceridemia, mixed hyperlipidemia, and hyperchylomicronemia; ICD-10-CM: E78.1, E78.2, E78.3, respectively). Hospitalizations were included beginning in 2016 as this is when ICD-10-CM codes became available for the Healthcare Cost and Utilization Project. Hospitalizations were excluded if the patient had idiopathic, biliary, alcohol-induced, or drug-induced etiologies (ICD-10-CM: K85.0x, K85.1x, K85.2x, and K85.3x, respectively).

### Outcomes

For each hospitalization meeting inclusion criteria, we extracted in-hospital death, LOS, patient charges which we converted to hospital cost, and whether the patient had a routine discharge to home. Hospital cost was calculated using NIS-provided cost-to-charge ratio files based on Centers for Medicare & Medicaid Services (CMS) cost reports; all costs were inflation-adjusted to 2020 US dollars [14]. The yearly rate of HTG-AP hospitalizations was estimated per 100,000 adult hospitalizations in the USA in which the NIS-estimated number of HTG-AP hospitalizations was divided by the Hispanic or non-Hispanic white resident adult population based on July 1st estimates provided by the United States Census Bureau and then multiplied by 100,000 [15, 16].

### Covariates

For each hospitalization, we extracted patient characteristics that included age, biological sex, primary payer (Medicare, Medicaid, private, other), income quartile, and transfer status, as well as the 29 Elixhauser comorbidities from which we calculated the validated Elixhauser comorbidity index (ECI) for in-hospital mortality (ECI scores range from − 19 to + 89; higher scores indicate a greater comorbidity burden) [17]. Although the NIS contains an Elixhauser comorbidity for diabetes status, we used diagnosis codes to identify type I diabetes (T1DM) (ICD-10-CM: E10.x and O24.0x) from type II diabetes (T2DM) (ICD-10-CM: E11.x and O24.1x). Further, we identified pregnancy status for each hospitalization in which the patient was female (ICD-10-CM: Z33.x, Z34.x, Z36.x, Z3A.x, and O09.x) as pregnancy can cause secondary hypertriglyceridemia. Finally, we identified whether plasmapheresis (PEX) was performed during the hospitalization (ICD-10-PCS: 6A550Z3, 6A551Z3).

### Statistical Analyses

All descriptive statistics are stratified by whether the patient was Hispanic or non-Hispanic white. Continuous variables are presented as median and interquartile range, whereas categorical variables are presented as percent; all presented descriptive statistics are based on weighted estimates. Continuous variables were compared between cohorts using a linear regression model, whereas categorical variables were compared using the Rao-Scott chi-square test. Year-over-year trend analyses were conducted using orthogonal polynomial contrasts with differences in trends evaluated via an interaction with year. Differences in in-hospital mortality and discharge to home were evaluated using unadjusted and adjusted logistic regression models, whereas LOS and hospital cost were compared using unadjusted and adjusted lognormal regression models. The adjusted models included age, biological sex, primary payer, income quartile, T1DM, T2DM, type of HTG (primary, mixed, hyperchylomicronemia), and alcohol abuse (defined by Elixhauser comorbidity). All analyses were conducted using SAS v. 9.4, accounted for the NIS sampling design, and used two-tailed *p* < 0.05 to indicate statistical significance.

## Results

### AP Hospitalizations

**Fig. 1:**
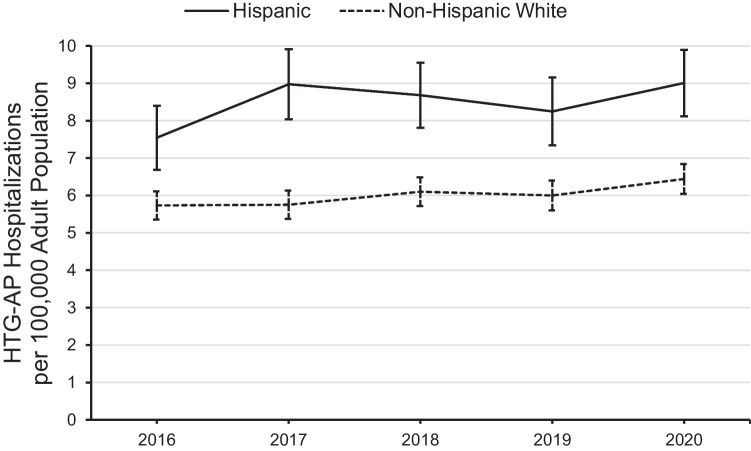
Year-over-year trend in the rate of HTG-AP hospitalizations in which the patient was Hispanic or non-Hispanic white. Error bars represent 95% confidence intervals. The linear trend for both Hispanics and non-Hispanic whites was statistically significant (both *p*_trend_ < 0.001); the increasing linear trends were statistically similar (trend interaction *p* = 0.860)

From 2016 through 2020, there were an estimated 1,387,669 hospitalizations for AP in the USA (95% CI 1,372,700 to 1,402,639) which represented an estimated 0.93% of adult hospitalizations in the USA during that period (95% CI, 0.93 to 0.94%). Of the AP hospitalizations, an estimated 5.9% were due to HTG-AP (95% CI, 5.8 to 6.0%; Table 1). Of those HTG-AP hospitalizations, an estimated 17,440 (22.1%, 95% CI, 21.3 to 22.9%) included a Hispanic patient and 48,235 (61.0%, 95% CI, 60.1 to 61.9%) included a non-Hispanic white patient. As shown in Fig. 1, the yearly rate of HTG-AP hospitalizations per 100,000 adult population was consistently and statistically higher for Hispanics compared to non-Hispanic whites. Further, the HTG-AP hospitalization rate increased for both Hispanics and non-Hispanic whites from study period start to finish (both *p*_trend_ < 0.001); however, the trends for Hispanics and non-Hispanic whites were not statistically different (interaction *p* = 0.860).

**Table 1:** Etiology of AP

	All hospitalizations (*N* = 1,387,669)	Hispanic (*n* = 174,630)	Non-Hispanic white (*n* = 865,310)
Hypertriglyceridemia	5.9	10.0	5.6
Idiopathic	4.0	2.7	4.6
Biliary	17.2	23.9	17.3
Alcohol-induced	26.9	21.4	25.6
Drug-induced	1.7	1.4	1.7
Other	2.1	1.8	2.2
Unspecified	42.2	38.8	42.9

Data presented as percent

### Baseline Characteristics

Table 2 shows the demographic and clinical characteristics of patients within HTG-AP hospitalizations. Demographically, Hispanics were younger, slightly more likely to be female, more commonly Medicaid recipients, and from zip codes with lower income quartiles. Clinically, Hispanics had higher rate of pure HTG and T2DM, with a similar rate of PEX and similar comorbidity burden; no pregnancies were identified.

**Table 2:** Demographic and clinical characteristics of HTG-AP hospitalizations in which the patient was Hispanic or non-Hispanic white

	Hispanic (*n* = 17,440)	Non-Hispanic white (*n* = 48,235)	*p*
Age	38 [31, 46]	46 [37, 55]	< 0.001
18–34	34.8	17.6	< 0.001
35–44	33.6	28.0	
45–54	21.0	26.7	
55–64	7.1	15.6	
65 +	3.4	12.1	
Biological sex			
Male	58.0	62.0	< 0.001
Female	42.0	38.0	
Primary payer			
Medicare	7.8	21.8	< 0.001
Medicaid	34.4	20.1	
Private	32.6	46.5	
Other	25.3	11.6	
Income quartile			
I	36.7	28.4	< 0.001
II	28.4	29.8	
III	22.5	25.0	
IV	12.2	16.9	
Hypertriglyceridemia			
Pure	92.6	85.2	< 0.001
Mixed	9.4	17.1	< 0.001
Hyperchylomicronemia	0.7	0.8	0.472
Diabetes			
Type I	4.7	4.0	0.108
Type II	65.8	58.4	< 0.001
Elixhauser comorbidity index	3 [–2, 9]	2 [–3, 9]	0.118
Transferred to different facility	2.9	5.0	< 0.001
Plasmapheresis	2.7	2.4	0.428

Data presented as median [IQR] or percent. All estimates are at the national level (i.e., weighted)

### Outcomes

Outcome data are presented in Table 3. Within HTG-AP hospitalizations, the number of observed in-hospital deaths within Hispanics was too low to report per the NIS Data Use agreement, which precluded any subsequent analysis; the in-hospital mortality rate for non-Hispanic whites was 0.4% (95% CI, 0.3 to 0.6%). Although hospital LOS was 4.7% longer for Hispanics (95% CI, 0.4 to 9.1% longer, *p* = 0.031; 3.4 days vs. 3.2 days), this difference was clinically negligible and became non-statistically significant after adjusting for demographic and clinical characteristics (adjusted days, 3.3 vs. 3.2, *p* = 0.175). However, both unadjusted and adjusted hospital costs were higher for Hispanics; specifically, costs were 18.9% higher for Hispanics compared to non-Hispanic whites (95% CI, 15.4 to 22.6% higher, *p* < 0.001; $9034 vs. $7606). Extrapolating this $1428 difference in adjusted hospital cost across all 17,440 HTG-AP hospitalizations in which the patient was Hispanic resulted in excess cost burden of $24,904,320 (or $4,980,864 annually) compared to non-Hispanic whites. Finally, Hispanics had 34.8% higher adjusted odds of routine discharge compared to non-Hispanic whites (95% CI, 16.4 to 56.0% higher, *p* < 0.001).

**Table 3:** Outcomes

	Unadjusted				Adjusted	
	Hispanic (*n* = 17,440)	Non-Hispanic white (*n* = 48,235)	Ratio (95% CI)	*p*	Ratio (95% CI)	*p*
In-hospital death	*	0.4	*	*	*	*
LOS	3.4	3.2	1.05 (1.01–1.09)	0.031	1.03 (0.99–1.08)	0.175
Hospital cost (2020 US$)	$9016	$7479	1.21 (1.17–1.24)	< 0.001	1.19 (1.15–1.22)	< 0.001
Discharge disposition						
Routine	92.2	88.5	1.54 (1.34–1.77)	< 0.001	1.35 (1.16–1.56)	< 0.001
Transfer	2.9	5.1				
Home health care	2.7	3.8				
Against medical advice	2.2	2.6				

An * indicates that the result cannot be presented due to small observed hospitalizations per the NIS Data Use Agreement. Hospital cost was inflation-adjusted to 2020 US dollars. The ratio for discharge disposition compared routine discharge vs. non-routine discharge (transfer, home health care, left against medical advice, and in-hospital death). Any ratio greater than 1 indicates that Hispanics had more of an outcome compared to non-Hispanic whites. The adjusted models included age, biological sex, primary payer, income quartile, type I diabetes, type II diabetes, type of hypertriglyceridemia (primary, mixed, hyperchylomicronemia), and alcohol abuse

## Discussion

To the best of our knowledge, this is the first study using the NIS to compare HTG-AP hospitalizations in Hispanic and non-Hispanic white. We found HTG-AP hospitalizations between 2016 and 2020 were more likely among Hispanic patients compared to non-Hispanic white patients (10% vs. 5.6%). Our demographic results are corroborated by the findings from a systematic review by Carr et al. [17]. Most patients with HTG-AP present in their 40 s; however, after we performed descriptive statistics, there was a notable 8-year difference between the mean ages of Hispanic and non-Hispanic whites with HTG-AP which has not been previously identified. Hispanic patients were also on average more likely female, from lower income quartile, and more likely to have T2DM and have Medicaid compared to non-Hispanic white patients. Despite clinically similar LOS, Hispanic patients sustained an average $1428 additional cost per HTG-AP hospitalization compared to non-Hispanic whites. When extrapolated across all 17,440 HTG-AP Hispanic hospitalizations, this amounts to an excess cost of approximately $24,904,320 across our 2016 to 2020 study period.

Our review of the NIS from 2016 to 2020 identified HTG-AP as the cause of an AP hospitalization in 5.9% of AP hospitalizations. Gapp et al. reviewed AP hospitalizations from 2001 to 2014 and found 3.7% of all AP hospitalizations were due to HTG [3]. Our findings suggest HTG-AP hospitalizations are becoming more frequent in the USA; a similar trend has been recently noted in other countries [4, 5]. As HTG-AP becomes more common, it is important to identify at risk groups with the goal of implementing preventative measures to lower disease burden and costs. The statistically higher yearly rate of HTG-AP hospitalizations could be due to differences in socioeconomic factors, access to healthcare, and provider availability. In their review of all-payer inpatient discharge databases, Feng et al. found that the preventable hospitalization rate among Hispanics was 13% higher than among Whites. These differences were mitigated when adjusting for socioeconomic status, healthcare access, and provider availability [18]. We found HTG-AP hospitalizations accounted for 10% of all AP hospitalizations in our Hispanic cohort. We do appreciate that ethnicity and race are social constructs and are not implying that these studies show intrinsic biological differences. We are also aware that the term “Hispanic” is inherently arbitrary and fluid but provides rough but valuable information about disease prevalence and risk factors. Our findings regarding biological sex are similar to the results from prior studies; men are more likely than women to develop HTG-AP [17]. Patients with familial HTG are at increased risk of having HTG-AP during the third trimester of pregnancy [7]. We evaluated the NIS for demographic data regarding pregnancy status but did not identify patients with HTG-AP and pregnancy. Prior studies suggest HTG-AP may be related to both bio-geographical ancestry, or a heritable component of ethnicity, and social determinants of health such as access to primary care, appropriate diabetes care [19–21]. Studies show that structural or social determinants of health strongly influence both disease incidence and outcomes for multiple medical conditions. Ethnicity and bio-geographical heritage are also associated with health outcomes.

Despite clinically similar LOS, hospital costs were 18.9% higher for Hispanic patients. Interpreter utilization does not explain these striking cost differences as prior studies showed per-patient interpreter fees do not exceed $300 per year [22]. We did not find a significant difference in PEX usage between our two cohorts. Notably, Hispanic hospitalization costs were higher despite their younger age. Hispanic patients did have more T2DM compared to non-Hispanic whites. Esparza et al. found T2DM to be one of the major conditions requiring aggressive treatment in patients with very severe HTG [23]. A 2019 study by Song et al. found the cost of 24 h of 120 units of regular insulin to be roughly $36 [24]. While length of stay was not significantly different between cohorts, it is possible patients with T2DM require more insulin infusions, more frequent monitoring, and a higher level of nursing care compared to those without T2DM. The difference in cost for insulin and nursing care may account for the hospital cost differences observed. The 38.5% higher adjusted odds of routine discharge noted among Hispanic patients is likely due to age differences between cohorts, as older patients are less likely to be discharged to home.

Our study has several limitations. Although the NIS provides a large, nationally representative sample of hospitalizations, it has limited ability to identify specific exclusion or inclusion criteria (e.g., triglyceride level is not available; thus, a homogenous criterion for HTG diagnosis is not possible). No validated set of ICD-10-CM diagnosis codes for HTG-AP exists. Therefore, HTG-AP hospitalizations were determined by excluding other common etiologies and diagnosis codes for unspecified acute pancreatitis (K85.9x) and other acute pancreatitis (K85.8x). Although we cannot determine for certain if all included patients had HTG-AP, when comparing the prevalence of HTG-AP hospitalizations of 5.9% to all of those for acute pancreatitis recorded in the NIS, our incidence rate approximates the accepted prevalence HTG-AP, approximately 9% [17]. HTG-AP has been associated with more severe AP and noted to carry increased mortality compared to other causes [25, 26]. The mortality rate for acute pancreatitis approximates 1%, but we found a mortality rate of 0.4% in the non-Hispanic white cohort [27]. Unfortunately, we could not compare mortality rates between the Hispanic and non-Hispanic white cohorts due to small sample size. This underrepresentation of mortality could be explained for by the transferred patients which accounted for between 2.9 and 5% of patients in each cohort; we could not report results when stratifying the transferred patients by ethnicity due to sample size limitations defined in the NIS data use agreement. Transferred patients are often transferred for a higher level of care and more likely to have higher mortality, and the outcome of these hospitalizations is likely underrepresented in the overall disposition breakdown [28].

Further, the NIS likely provides incomplete data on patient comorbidities as comorbidity identification is driven completely by discharge diagnosis codes which tend to be coupled with billing practices and not fully representative of the patients being studied [29]. Risk factors for hospitalization (e.g., medication adherence), a thorough family history (which may reveal familial HTG and an increased risk of pancreatitis), and severity of presenting illness could represent unaccounted confounding variables. Additionally, the NIS does not distinguish ethnicity and race when the data variable noting Hispanic ethnicity is assigned. Therefore, comparison of non-white Hispanics and white-Hispanics could not occur. This is an important limitation as Hispanics are heterogeneous with differences across subgroups in sociocultural practices, environmental exposures, genetic backgrounds, and cultural histories that shape their predispositions to develop chronic diseases [30]. There are limitations in using the NIS to describe disparities along race/ethnicity categories due to missing or misclassified data, given subgroup analysis based on ethnicity and national origin could not be performed.

In conclusion, our retrospective study of HTG-AP hospitalizations using NIS data identified ethnic disparities in patient charges with Hispanics incurring greater hospital costs per hospitalization than non-Hispanic whites despite clinically similar comorbidity burden and LOS. The reason for the noted cost disparities is unclear but may be contributed by higher rates of T2DM among Hispanic patients. Nationally, Hispanics hospitalized with HTG-AP tended to be younger. These results are concerning given the recurrent and more severe clinical course associated with HTG-AP compared to pancreatitis caused by other etiology [31, 32]. Physician awareness regarding these striking disparities in HTG-AP will hopefully galvanize efforts to optimize HTG and hyperglycemia therapy especially in Hispanic patients. Additionally, if patients are aware of their risk and propensity for increased morbidity and cost, patients may be incentivized to adopt preventative strategies. A more complete understanding of differences in structural determinants of health that lead to disparities between Hispanics and non-Hispanic whites will help achieve healthcare equity in HTG-AP. Future efforts should focus on identifying barriers to proper outpatient follow-up, continuation of triglyceride-lowering therapy, HTG-AP and pregnancy in Hispanic patients, and the comparative burden of readmissions for HTG-AP based on race and ethnicity.

## Data Availability

Data are available for purchase from the HCUP NIS website.

## Abbreviations

HTG-AP: Hypertriglyceridemia-induced acute pancreatitis
NIS: National Inpatient Sample
HCUP: Healthcare Cost and Utilization Project
LOS: Length of stay
PEX: Plasmapheresis

## Code Availability

SAS code is available upon request.
